# Cofactor analogue-induced chemical reactivation of endonuclease activity in a DNA cleavage/methylation deficient TspGWI N_473_A variant in the NPPY motif

**DOI:** 10.1007/s11033-014-3085-x

**Published:** 2014-01-19

**Authors:** Agnieszka Zylicz-Stachula, Joanna Jeżewska-Frąckowiak, Piotr M. Skowron

**Affiliations:** Department of Molecular Biotechnology, Institute for Environmental and Human Health Protection, Faculty of Chemistry, University of Gdansk, Wita Stwosza 63, 80-952 Gdansk, Poland

**Keywords:** Endonuclease-methyltransferase, *Thermus* sp. enzyme, Enzymatic reaction cofactor, Cofactor analogue, Sinefungin, *S*-adenosylmethione, Mutant activation, Specificity change

## Abstract

We reported previously that TspGWI, a prototype enzyme of a new *Thermus* sp. family of restriction endonucleases-methyltransferases (REases-MTases), undergoes the novel phenomenon of sinefungin (SIN)-caused specificity transition. Here we investigated mutant TspGWI N_473_A, containing a single amino acid (aa) substitution in the NPPY motif of the MTase. Even though the aa substitution is located within the MTase polypeptide segment, DNA cleavage and modification are almost completely abolished, indicating that the REase and MTase are intertwined. Remarkably, the TspGWI N_473_A REase functionality can be completely reconstituted by the addition of SIN. We hypothesize that SIN binds specifically to the enzyme and restores the DNA cleavage-competent protein tertiary structure. This indicates the significant role of allosteric effectors in DNA cleavage in *Thermus* sp. enzymes. This is the first case of REase mutation suppression by an S-adenosylmethionine (SAM) cofactor analogue. Moreover, the TspGWI N_473_A clone strongly affects *E. coli* division control, acting as a ‘selfish gene’. The mutant lacks the competing MTase activity and therefore might be useful for applications in DNA manipulation. Here we present a case study of a novel strategy for REase activity/specificity alteration by a single aa substitution, based on the bioinformatic analysis of active motif locations, combining (a) aa sequence engineering (b) the alteration of protein enzymatic properties, and (c) the use of cofactor–analogue cleavage reconstitution and stimulation.

## Introduction

With the advent of bioinformatics, research into the restriction-modification (RM) of DNA received a strong impulse, both to further evaluate basic research aspects and develop new tools for genetic engineering. After reaching a relative plateau over 10 years ago in the search for new prototype specificities using the classic biochemical method, rapid bacterial genome and metagenomic sequencing coupled with homology, evolutionary and advanced active motif software predictions has currently opened up a new chapter in the efficient search for gene candidates for new RM as well as structure–function and DNA sequence-protein folding analysis [1–5]. Sequence-structure–function bioinformatics studies provide data for the controlled sequence-specific protein specificity/activity determination of functionally critical aa as well as rational protein engineering. In contrast to the majority of proteins, a significant sequence similarity between REases is extremely rare, even when isoschizomers are compared. Recent advances relating to the construction of a homology model MwoI with DNA have pinpointed functionally important aa that have subsequently been targeted by mutagenesis, resulting in changes in enzyme specificity [6]. With the discovery of new genes and enzymes, numerous atypical REases have been discovered in recent years, which depart from established paradigms, such as the radically different magnesium-independent BfiI REase. This evolved by an entirely different route than other REases, emerging as a fusion protein between a Mg^2+^-independent non-specific nuclease and a B3-like DNA-binding domain from plant transcription regulatory protein [7]. Interestingly, bioinformatics studies have shown that a BfiI homologue exists in *Mesorhizobium* sp. bacteria BNC, which is a symbiont of a plant capable of growing in an EDTA-rich environment [7]. Another natural REase – CviJI of unusual eukaryotic origin, coded by IL-3A virus infected *Chlorella* algae—is an adenine nucleotide-stimulated enzyme (a feature not found among other natural REases) that cleaves a 2–3 bp cognate site. It is therefore the most frequently recognized DNA REase among the over 300 prototypes known [8–10]. We have constructed two chemically modified, frequently cleaving artificial REases, namely TspGWI/SIN, an alternative to CviJI, cleaving the 3-bp averaged prototype site [11], and TaqII/SIN/DMSO, cleaving the 2.9-bp averaged prototype site [12, 13].

Both TspGWI and TaqII belong to the *Thermus* sp. enzyme family—another atypical group of REases, which we have defined [11–17, 19]. The existing members of the family include six related thermostable enzymes: TspGWI [ACGGA (11/9)] [15, 16], TspDTI [(ATGAA (11/9)] [14, 17], Tth111II/TthHB27I [(CAARCA (11/9)] [14, 17, 18], TsoI [TARCCA (11/9)] [10, 17, 19] and TaqII [(GACCGA (11/9) or CACCCA (11/9)] [12, 20]. Recent analysis and literature data have shown the existence of putative or partially analysed members (or genes) of the *Thermus* sp. family, originating from evolutionarily distant mesophilic bacteria [10, 19]. This may indicate two possible routes by which the family evolved: (*i*) divergent evolution following horizontal interspecies transfer from a common ancestor of the family, or (*ii*) the formation of an enzyme pro-prototype even earlier in a single bacteria species and further proliferating its specificity and other features as the host evolves and gives origin to a new bacterial species/strains.

In this paper we describe further studies on the TspGWI bifunctional REase, showing a novel type of mutation suppression induced by a cofactor analogue, where the NPPY methylation catalytic motif is converted to a non-functional APPY segment, while the restriction activity becomes reactivated in vitro.

## Materials and methods

### Bacterial strains, plasmids, media and reagents

A wild-type (wt), recombinant TspGWI protein expression plasmid (pRZ-TspGWI) was constructed previously [16]. Site-directed mutagenesis within the *tspGWIRM* gene to make pRZ-TspGWI APPY was described previously [16]. The procedures of recombinant wt TspGWI clone culturing, induction and protein purification were amended for the properties of the TspGWI N_473_A variant (NPPY motif to APPY transition) in order to ensure that thermostable protein folding conditions were maintained. Marathon DNA Polymerase was from A&A Biotechnology (Gdansk, Poland), and T7 bacteriophage DNA, plasmids pBR322 were from Vivantis Technologies (Shah Alam, Malaysia).The PCR primer synthesis was performed at Genomed (Warsaw, Poland). All other reagents were purchased from Sigma-Aldrich (St Louis, MO, USA). 100 bp DNA and 1 kb DNA markers were from Fermentas (Thermo Fisher Scientific, MA, USA).

### Expression of the *tspGWIRM* (N_473_A) gene under control of the *P*_*R*_ promoter in *E. coli* and purification of the TspGWI N_473_A enzyme

**Fig. 1:**
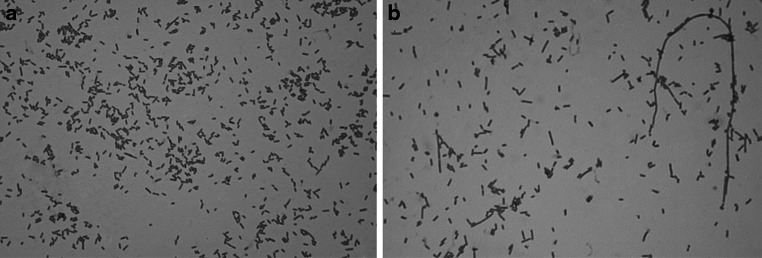
Microscope imaging of methylene blue stained *E. coli* cells harbouring wt *tspGWIRM* or *tspGWIRM* *N*_*473*_ *A* gene. Samples of the bacterial culture were taken both from *E. coli* cells expressing wt TspGWI and TspGWI N_473_A variant, 4 h after promoter *P*_*R*_ transcription induction. After standard methylene blue positive staining, slide glasses preparations were observed under Olympus CX21FS1 light microscope with total ×1200 magnification

*Escherichia coli* DH11S [pRZ-TspGWI APPY] was expressed in TB medium supplemented with chloramphenicol (40 μg/ml) and maltose (0.5 %) at 30 °C with vigorous aeration, followed by *P*_*R*_ promoter induction as a result of a temperature shift to 42 °C. Simultaneously, expression of the wt *tspGWIRM* clone was performed, under the same conditions, for growth and induction control purposes. Expression of *tspGWIRM* (N_473_A) in *E. coli* DH11S [pRZ-TspGWI APPY] was initiated with bacterial inoculum flushed from a Petri dish into 500 ml of medium. The culture was grown with vigorous aeration until OD_600_ reached 0.6, and the temperature shift was performed by the addition of 500 ml of medium pre-warmed to 60 °C. The culture was further supplemented with chloramphenicol. Uninduced control and induced cells were subjected to 10 % SDS-PAGE, and gels were analysed for the appearance of the expected polypeptide size of app. 120 kDa and for endonucleolytic activity in crude lysates (recombinant wt TspGWI only). The recombinant wt TspGWI and TspGWI N_473_A proteins concentrations were determined utilizing Coomassie Brillant Blue stained protein band densitometric analysis, with the use of BSA serial dilutions calibration curve. The concentration values were obtained from a linear standard reflective scan mode with background correction in UN-SCAN IT GEL for Windows 6.1 data software (v. 6.1, Gel Analysing and Graph Digitizing Software, Silk Scientific Corporation, Orem, UT, USA). Additionally, 4 h after induction, samples for microscopic analysis were taken from both recombinant wt TspGWI and TspGWI N_473_A mutant cultures. Bacteria were stained following the standard methylene blue positive staining protocol [21]. Slide glasses preparations were observed and photographed with immerse objective lenses of 100× magnification, under an Olympus CX21FS1 light microscope with a total obtained magnification of 1200× (Fig. 1). The culture growth was continued for 14 h at 42 °C; both variants of TspGWI were accumulating slowly, becoming detectable after only 2 h. The purification scheme varied from the one we described previously for the native (*Thermus* sp. GW-purified) and recombinant wt TspGWI (*E. coli*-purified) enzymes [16], and included the following stages:

1. Polyethyleneimine (PEI) removal of nucleic acids from a sonicated and centrifuged bacterial extract, obtained by suspension of 16 g of cells in 4 volumes of buffer A [20 mM Tris–HCl (pH 8.0 at 25 °C), 0.5 mM EDTA, 50 mM NaCl, 5 % glycerol, 10 mM 2-mercaptoethanol (ßMe), 1 mM PMSF, 1 mg/ml lysozyme], addition of NaCl to 400 mM concentration, and the gradual addition of PEI to 0.4 %. Following 30 min. of stirring, the sample was centrifuged and the supernatant subjected to ammonium sulphate (AmS) fractionation.
2. AmS fractionation was conducted in two phases. In the first step, 35 % saturation (at 4 °C, 0.208 g/ml), contaminating proteins were removed. In the second stage, 50 % saturation was applied (additional 0.094 g/ml), the suspension stirred overnight, centrifuged, dissolved in buffer B and dialysed against buffer B [20 mM K/PO_4_ (pH 8.0 at 25 °C), 0.5 mM EDTA, 50 mM NaCl, 0.02 % Triton X-100, 0.02 % Tween 20, 5 % glycerol, 10 mM ßMe,1 mM PMSF].
3. Phosphocellulose chromatography was conducted in buffer B and the proteins eluted with the following NaCl steps in buffer B (mM): 100, 200, 300, 400, 500 and 1 M. TspGWI variants were dialysed against buffer C [20 mM Tris–HCl (pH 8.0 at 25 °C), 0.5 mM EDTA, 30 mM NaCl, 0.01 % Triton X-100, 0.01 % Tween 20, 5 % glycerol, 10 mM ßMe, 0.1 mM PMSF]. TspGWI variants eluted at 200–300 mM NaCl.
4. DEAE-Sephadex chromatography using buffer C employed NaCl steps in buffer C (mM): 100, 150, 200, 250, 300, 350, 400, 450 and 500. TspGWI variants eluted at 150–200 mM NaCl. The DEAE-Sephadex chromatography was repeated twice. Pooled column fractions containing the enzyme were dialysed against buffer C between repeated procedures and finally against buffer D [20 mM K/PO_4_ (pH 7.0 at 25 °C), 100 mM NaCl, 0.01 % Triton X-100, 5 % glycerol, 10 mM ßMe, 0.1 mM PMSF].
5. Hydroxyapatite chromatography was used to adsorb TspGWI proteins, the column was flushed with buffer D, and the following K/PO_4_ buffer steps were used for elution (mM): 40, 80, 120, 160, 200, 240 and 280. TspGWI variants eluted at 150–200 mM K/PO_4_. Final preparations were dialysed against storage buffer P [20 mM Tris–HCl (pH 8.3 at 25 °C), 0.1 mM EDTA, 25 mM KCl, 40 mM AmS, 0.05 % Tween 20, 0.5 mM DTT, 50 % glycerol].

**Fig. 2:**
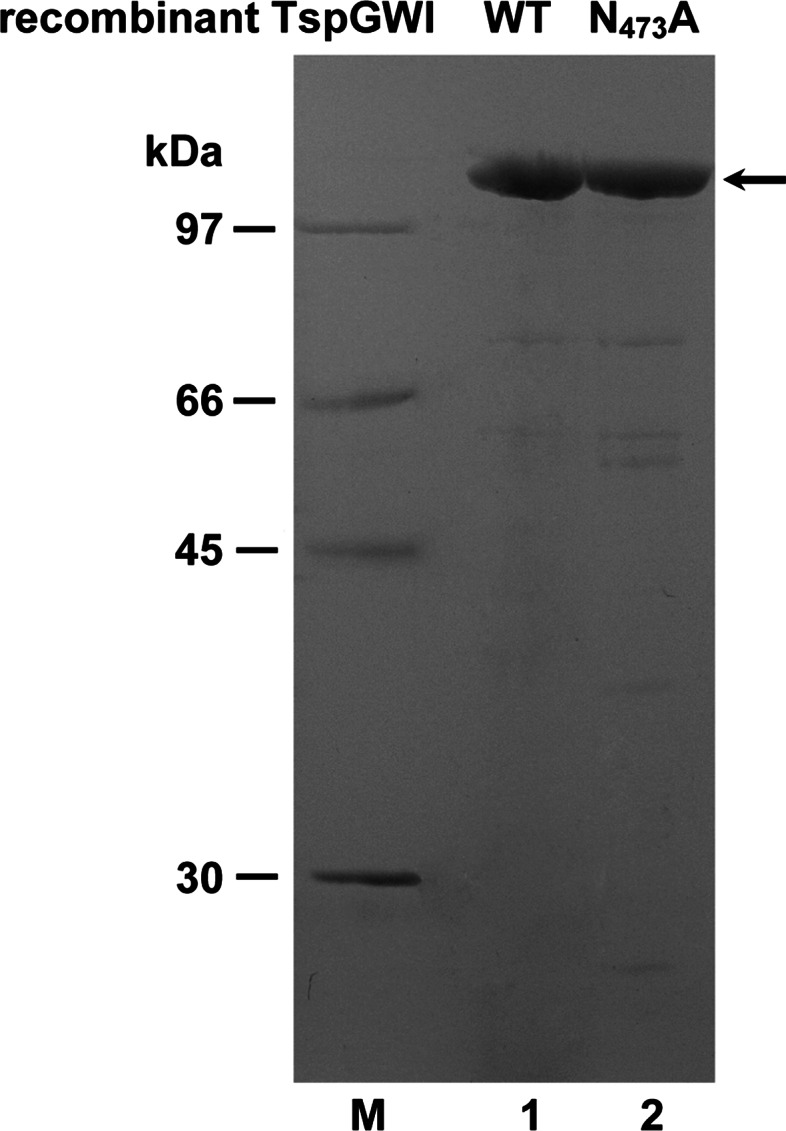
SDS-PAGE analysis of purified wt recombinant TspGWI and TspGWI N_473_A proteins. Purified protein preparations containing 2 μg of wt recombinant TspGWI or TspGWI N_473_A were electrophoresed in 10 % SDS-PAGE. Lane M, protein marker (GE Healthcare); lane 1, purified protein preparation containing 2 μg of wt recombinant TspGWI REase; lane 2, purified protein preparation containing 2 μg of TspGWI N_473_A variant. Both TspGWI variants are indicated with an *arrow*

The purity of both enzyme preparations was estimated on a 10 % SDS-PAGE gel. Loaded samples contained 2 μg of wt or mutant TspGWI proteins (Fig. 2).

### REase activity in DNA cleavage assay

**Fig. 3:**
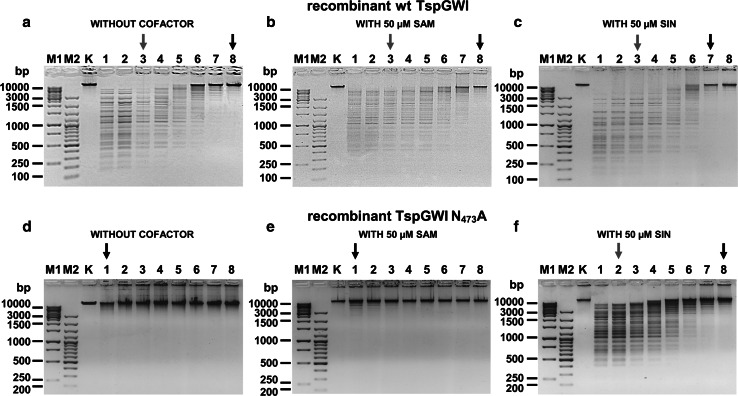
T7 bacteriophage DNA cleavage patterns of TspGWI protein variants in the presence or absence of SAM and its analogue SIN. 300 ng of T7 bacteriophage DNA (0.99 pmol TspGWI recognition sites) was digested for 1 h at 65 °C with consecutive two-fold REase dilutions, starting from 500 ng (4.16 pmol; 4.2:1 molar ratio of enzyme to recognition sequence) down to 3.91 ng (33 fmol; 0.03:1 molar ratio of enzyme to recognition sequence) of either TspGWI or TspGWI N_473_A variant in buffer T, without SAM/SIN or supplemented with 50 μM of the SAM or SIN. The *black arrows* indicate the lanes of estimated identical or nearly identical extent of DNA digestion. The *red arrows* indicate lanes with a stable partial digestion pattern, obtained using the minimum sufficient amount of an enzyme. **a** Cleavage pattern of recombinant wt TspGWI in the absence of SAM and SIN. Lanes M, GeneRuler™ 1 kb DNA Ladder (Thermo Fisher Scientific), selected bands marked; M2 GeneRuler™ 100 bp DNA Ladder (Thermo Fisher Scientific), selected bands marked; lane K, untreated T7 bacteriophage DNA; lane 1–8, T7 DNA cleaved with the wt TspGWI protein, two-fold serial dilutions series. The reaction products were resolved on 1.3 % agarose gel in TBE buffer and stained with ethidium bromide (EtBr). **b** As in Panel a, except that the reactions were supplemented with 50 μM of SAM. **c** As in Panel a, except that the reactions were supplemented with 50 μM of SIN. **d** As in Panel a, except that the reactions were carried out with the TspGWI N_473_A variant. **e** As in Panel a, except that the reactions were carried out with the TspGWI N_473_A variant and supplemented with 50 μM of SAM. **f** As in Panel a, except that the reactions were carried out with the TspGWI N_473_A variant and supplemented with 50 μM of SIN

**Fig. 4:**
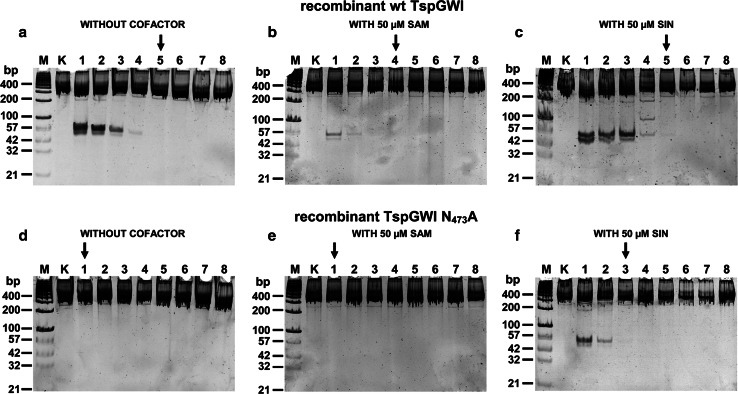
Comparative titration of TspGWI protein variants on the 390 bp PCR DNA substrate in the presence or absence of SAM or its analogue SIN. 300 ng of 390 bp custom PCR, containing two convergent 5′-ACGGA-3′ recognition sites (2.28 fmol TspGWI recognition sites), was digested with consecutive two-fold REase dilutions, starting from 500 ng (4.16 pmol; 1.82:1 molar ratio of enzyme to recognition sequence) down to 3.91 ng (33 fmol; 0.01:1 molar ratio of enzyme to recognition sequence) of recombinant wt TspGWI or TspGWI N_473_A variants in buffer T, supplemented with 50 μM of the SAM or SIN, for 1 h, at 65 °C. **a** Cleavage pattern of recombinant wt TspGWI in the absence of SAM and SIN. Lanes M, GeneRuler™ 100 bp DNA Ladder (Thermo Fisher Scientific), supplemented with four additional small size DNA marker fragments: 21, 32, 42 and 57 bp (selected bands marked); lane K, untreated PCR DNA; lane 1–8, PCR DNA cleaved with the recombinant wt TspGWI protein variant, two-fold serial dilution series. The reaction products were resolved on 15 % polyacrylamide gel in TBE buffer and stained with Sybr Green I. **b** As in Panel a, except that the reactions were supplemented with 50 μM of SAM. **c** As in Panel a, except that the reactions were supplemented with 50 μM of SIN. **d** As in Panel a, except that the reactions were carried out with the TspGWI N_473_A variant. **e** As in Panel a, except that the reactions were carried out with the TspGWI N_473_A variant and supplemented with 50 μM of SAM. **f** As in Panel a, except that the reactions were carried out with the TspGWI N_473_A variant and supplemented with 50 μM of SIN

**Fig. 5:**
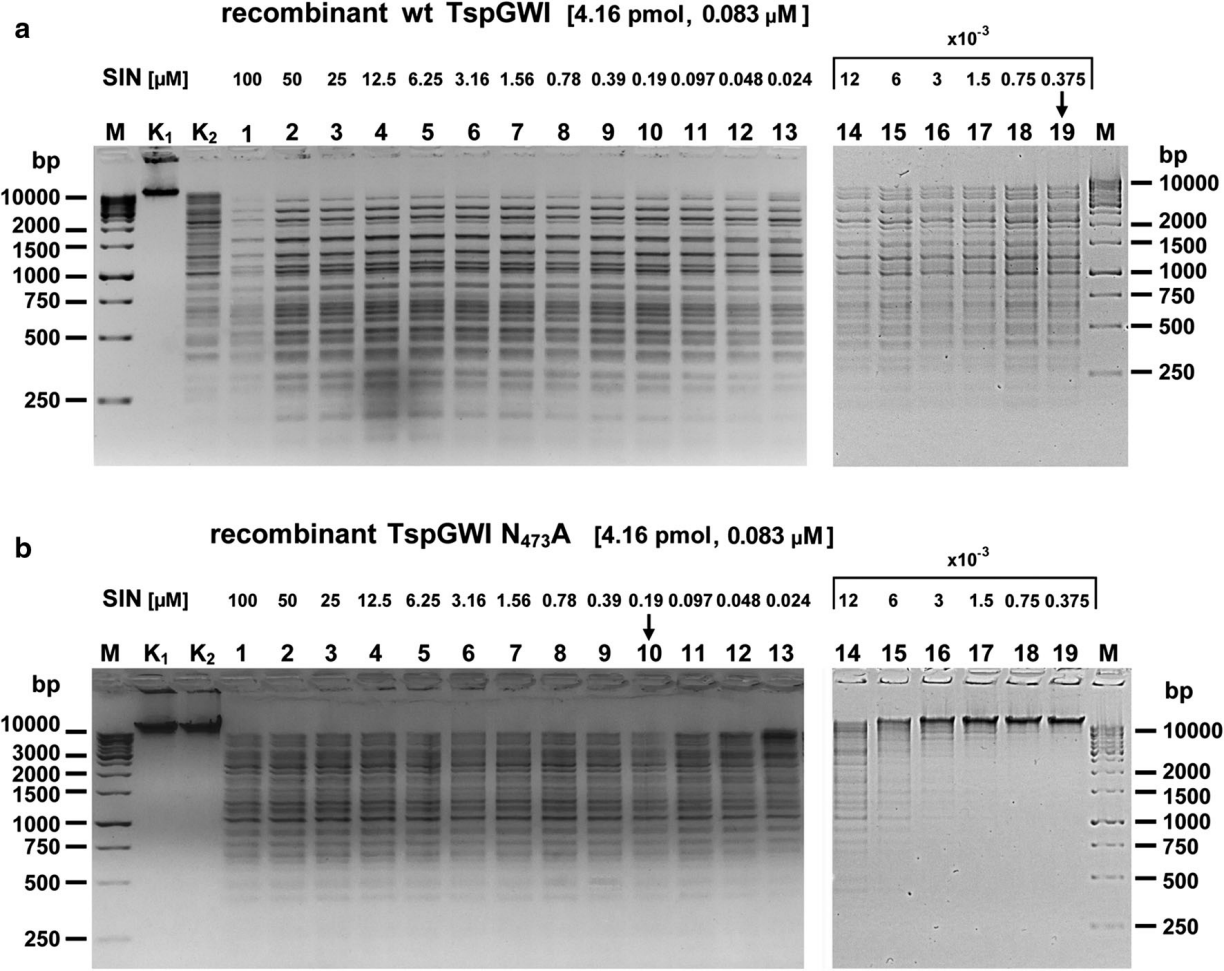
Comparative SIN titrations in the presence of wt TspGWI REase or TspGWI N_473_A variant protein. 300 ng of T7 bacteriophage DNA (0.99 pmol restriction sites) was digested with 500 ng (4.16 pmol; 4.2:1 molar ratio of enzyme to recognition sequence) of a protein variant TspGWI or TspGWI N_473_A in buffer T, supplemented with consecutive two-fold SIN dilutions, starting from 100 μM of the SIN down to 3.75 × 10^−3^ μM (0.375 nM), for 1 h at 65 °C. The *black arrow* indicates the first lane with lower than stoichiometric concentration of SIN. **a** Wt TspGWI-generated cleavage pattern in the presence of consecutive two-fold SIN dilutions. Lanes M, GeneRuler™ 1 kb DNA Ladder (Thermo Fisher Scientific), selected bands marked; lane K_1_, untreated T7 bacteriophage DNA; lane K_2_, T7 DNA cleaved with the wt TspGWI protein, without SIN; lane 1–19, T7 DNA cleaved with the recombinant wt TspGWI, supplemented with SIN, two-fold dilutions series. The reaction products were resolved on 1.3 % agarose gel in TBE buffer and stained with EtBr. **b** As in Panel a, except that the reactions were carried out with the TspGWI N_473_A variant

**Fig. 6:**
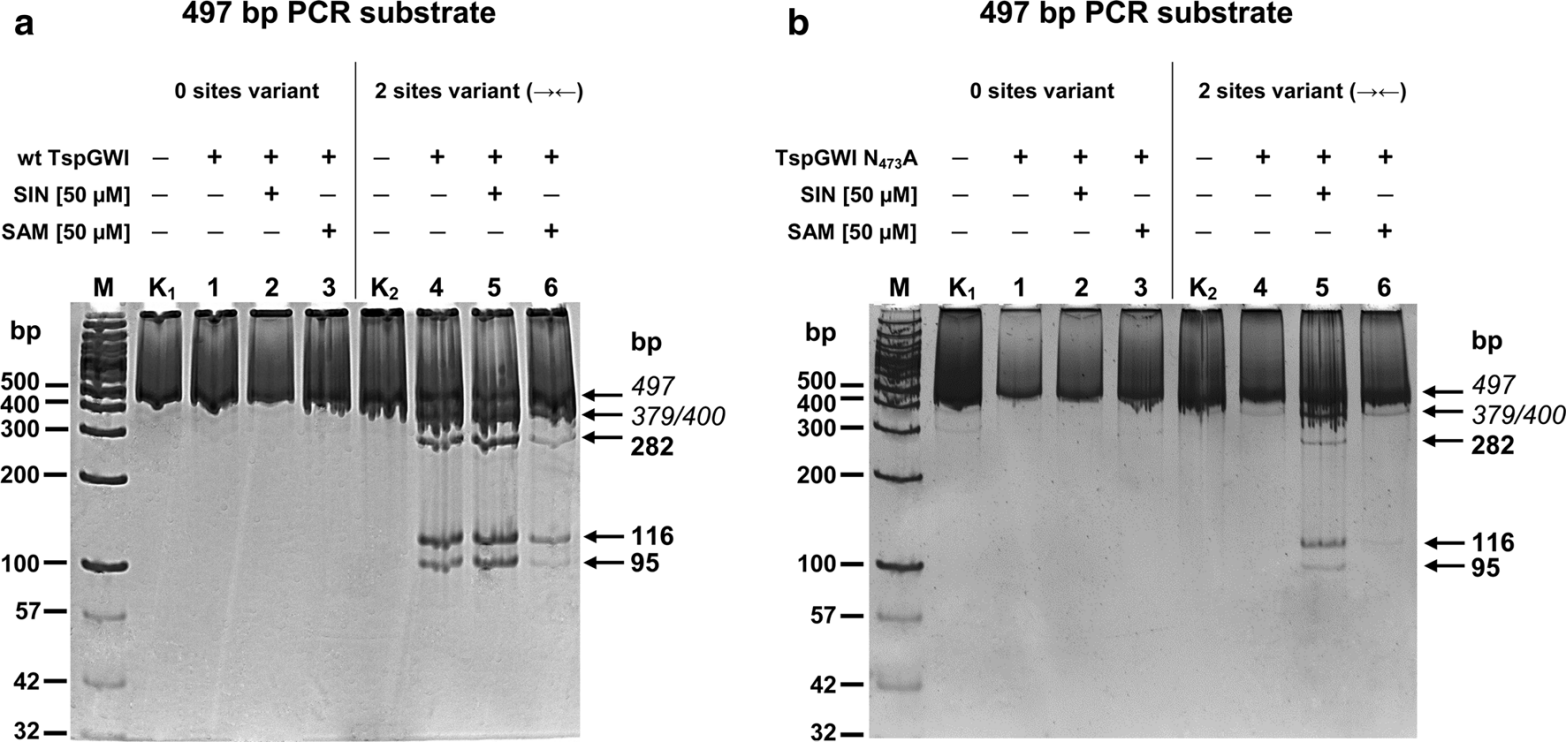
TspGWI N_473_A variant REase DNA cleavage specificity determination. 300 ng of 497 bp custom prolonged PCR, containing two convergent 5′-ACGGA-3′ recognition sites (2 sites variant (→←)), or having TspGWI recognition sites deleted (0 sites variant) were digested with 4.16 pmol of recombinant wt TspGWI or TspGWI N_473_A variants in buffer T, in the presence or absence of 50 μM SAM or SIN, for 1 h, at 65 °C. **a** Recombinant wt TspGWI cleavage pattern of 497 bp ‘zero’ or ‘2 sites’ variant PCR substrates. Lanes M, GeneRuler™ 100 bp DNA Ladder (Thermo Fisher Scientific), supplemented with DNA markers as in Fig. 4 (selected bands marked); lane K_1_, untreated 497 bp ‘zero sites’ variant PCR substrate; lane K_2_, untreated 497 bp ‘2 sites’ variant PCR substrate, lane 1–3, ‘zero sites’ variant 497 bp PCR DNA cleaved with the wt TspGWI protein variant, lane 4–6, ‘2 sites’ variant 497 bp PCR DNA cleaved with the wt TspGWI protein. The reaction products were resolved on 15 % polyacrylamide gel in TBE buffer and stained with Sybr Green I. **b** As in Panel a, except that the reactions were carried out with the TspGWI N_473_A protein variant

Standardized conditions for DNA cleavage were used, both in the presence and absence of 50 μM SAM (cofactor) or SIN (cofactor analogue), in an optimal reaction buffer T [50 mM Tris–HCl (pH 7.2 at 65 °C), 10 mM MgCl_2_,10 mM DTT] [15]. The reaction volume of 50 μl contained either 300 ng bacteriophage T7 DNA or 300 ng custom PCR substrate. All experiments utilized recombinant wt TspGWI or TspGWI N_473_A variant protein. Under those conditions both SAM and SIN exhibited sufficient stability (not shown). The titration experiments were carried out as a series of two-fold dilutions, starting from 500 ng (4.16 pmol) down to 3.91 ng (33 fmol) of wt TspGWI protein or TspGWI N_473_A mutant (Figs. 3, 4). SIN titration (Fig. 5) or cleavage specificity determination (Fig. 6) were performed in the presence of 4.16 pmol of wt TspGWI or TspGWI N_473_A. To precisely compare small differences in digestions extent, the reactions were carried out at enzyme/time-limiting conditions, thus avoiding overdigestion conditions. After 1 h at 65 °C the reactions were immediately stopped by the addition of EDTA, SDS, proteinase K and the REases were digested for 1 h at 55 °C, then the DNA was ethanol-precipitated. The DNA precipitate was collected by centrifugation and redissolved in 10 mM Tris–HCl (pH 8.0 at 25 °C). The custom 390 bp PCR fragment [11] was constructed for comparative titrations of TspGWI protein variants (Fig. 4). The PCR containing double convergent (→←) canonical sites for TspGWI was constructed using a pair of primers: 5′-CTCGACCTGAATGGAAGCCG-3′ and 5′-GGTGCAGGGCGCTGACTTCC-3′, amplifying a modified DNA segment from pBR322 plasmid. The TspGWI ‘zero sites’ custom 390 bp PCR variant was constructed using a pair of PCR mutagenic primers: 5′-CTCGACCTGAATGGAAGCCGGCGGCACCTCGCTGACCGATTCACCACT-3′ and 5′-GGTGCAGGGCGCTGACTTCCGCGTTTCCAGACTTTACGAAACACCCAAACCGAAGA-3'. For the purpose of clearer interpretation of the TspGWI N_473_A mutant cleavage specificity assay (Fig. 6), the distances from both 5′ and 3′ PCR ends to the TspGWI recognition sites were extended in both the ‘double convergent’ and ‘zero sites’ custom substrates, resulting in two, nearly identical 497 bp PCR fragments, with point bp changes within the TspGWI recognition sequences. The prolonging primers complementary to the 390 bp PCR were designated F63-390 and R75-390, as we described previously [12]. The electrophoresis samples were heated at 65 °C for 5 min. in a loading buffer, containing SDS and EDTA to facilitate DNA release from complexes with TspGWI protein variants. The PCR DNA cleavage products were analysed by 15 % polyacrylamide gel electrophoresis in TBE buffer, while T7 DNA cleavage products were resolved in a 1.3 % TBE agarose gel. Complete digestion of the 390 bp PCR product by TspGWI would yield 282 bp, 56 bp and 48 bp fragments. Complete digestion of the 497 bp PCR product by TspGWI would yield 282 bp, 116 bp and 95 bp fragments. DNA bands were quantitatively compared by applying UN-SCAN IT GEL for Windows 6.1 data software to a series of photographs taken with different exposure times.

## Results and discussion

The prototype TspGWI-coding gene was the first in a series of related genes from the *Thermus* sp. family cloned by our group [16], and was originally found in the very same thermal water sample as another prototype member of the family—TspDTI [14, 17]. Bioinformatics analysis of the *Thermus* sp. family RM genes coupled with experimental confirmation by site-directed mutagenesis [16] defined distinct functional regions, fused within a single polypeptide: Type I REases-like domains arranged in tandem. Conspicuous are the central HsdM-like module (helical domain), conserved MTase domain and *N*-terminal nuclease domain, similar to the corresponding domains in HsdR subunits. Both the ATP-dependent translocase module of the HsdR subunit and the supplementary domains involved in subunit–subunit interactions in Type I systems are missing. This indicates that structurally and functionally, the *Thermus* sp. enzyme protomers correspond to the streamlined ‘half’ of a Type I enzyme [16]. The structural and functional domain arrangement of TspGWI and other *Thermus* sp. family of REases was shown in our previous publications [16, 17].

With the use of bioinformatics analysis and preliminary site-directed mutagenesis studies we designed and constructed mutants in TspGWI polypeptide domain motifs, crucial to REase and MTase activities [16]. These included REase catalytic motif PD-EXK, the SAM binding motif DPAVGTG and the typical methylation catalytic motif NPPY. Somewhat surprisingly, further cloning, biochemical and bioinformatics studies revealed that both TspGWI and TspDTI, though belonging to the *Thermus* sp. family, are more remotely related to each other than to the other members found in distant hot spring/thermal water locations (separated by thousands of kilometres and/or ocean). TspGWI is similar to TaqII and TspDTI resembles Tth111II, so the family is divided into two sub-families: TspGWI-like [16] and TspDTI-like [17]. The differences relate not only to the general aa sequence similarity, but also to particularly active motifs. Enzymes from the TspGWI-subfamily—TspGWI, TaqII and the hitherto uncharacterized RpaI, originating from mesophilic bacteria [10, 19]—have typical REase catalytic motifs PD-(D/E)XK [16, 22], methylation catalytic NPPY and SAM binding motifs (DPA(V/M)GTG [16]. On the other hand, the enzymes from the TspDTI-subfamily—TspDTI, Tth111II/TthHB27I, TsoI and the so far uncharacterized CchII, originating from mesophilic bacteria [10, 19]—have atypical REase catalytic motifs D-EXE (also detected in typical Type II BamHI REase) [17], a less frequent NPPW methylation catalytic motif and cysteine+serine containing SAM binding motifs (D/P)PACGSG [17]. There is also another remarkable functional difference. We recently reported a new phenomenon of SIN-mediated specificity change (‘star affinity‘) in TspGWI from a 5-bp recognition site to an averaged 3-bp recognition site [11] and in TaqII from a 5–6-bp recognition site to an averaged 2.9-bp recognition site [12, 13]. From these studies conducted on wt proteins, we set up the hypothesis that SIN, being a chemical analogue of similar structure, is capable of mimicking SAM in the protein specific binding pocket, but is not a methyl group donor. Moreover, SIN possesses a reversed charge distribution as compared to SAM. The combination of these features may cause subtle conformational and functional changes in *Thermus* sp. REases’ tertiary structure upon the cofactor analogue binding and consequently alter DNA recognition [12, 13]. In contrast, none of the several TspDTI-like subfamily enzymes tested in our laboratory exhibited ‘star affinity’, thus this phenomenon is limited hitherto to enzymes possessing PD-(D/E)XK, NPPY and (DPA(V/M)GTG functional motifs [13, 17].

Investigating TspGWI mutants [16] in more detail, we found a combination of unusual biochemical features in one of the mutants: it contained a single aa substitution of asparagine to alanine residue in position 473 of its MTase polypeptide region. The TspGWI N_473_A, with the NPPY motif converted to APPY, confirmed the results of the bioinformatics analysis. This mutant had no MTase activity [16]. We anticipated serious difficulties in or even the impossibility of cultivating recombinant *E. coli* with this mutant clone, owing to the expected residual TspGWI REase activity, even though we used reduced cultivation temperatures (28–30 °C) and modified vectors to diminish thermostable REase activity in vivo. However, despite being more fragile (prone to lysis), growing more slowly than their equivalents with the wt TspGWI clone and exhibiting altered cell morphology, the mutant-carrying bacteria were successfully cultured (Fig. 1). Strikingly, the TspGWI N_473_A (Fig. 1b) expressing *E. coli* cells are much more different morphologically than those expressing wt TspGWI (Fig. 1a). The TspGWI N_473_A producing cells are noticeably longer and the culture population is heterogeneous. There is an observed frequent presence of filaments of variable length, including species which are 5 to over 100 (!)-fold longer than *E. coli* cells expressing the wt TspGWI protein (Fig. 1ab). Apparently, this MTase-deficient and active only partially REase mutant, even though it is a thermostable enzyme (thus less active at induction temperature of 42 °C), produces enough chromosomal cleavages, exercising a strong selective pressure on recombinant *E. coli* cells, to repair DNA breaks. On the other hand, the residual REase activity is low enough to be sub-lethal only, thus allowing cells to survive, by inhibiting cells divisions. Conclusion can be drawn that bacterial chromosome replication and cell division is being slowed down/stalled as a consequence of the necessity to repair TspGWI N_473_A-generated DNA cuts in vivo. As a result, the active TspGWI N_473_A clone takes control over recombinant *E. coli* division. Thus, it acts as a ‘selfish gene’. This would corroborate with Kobayashi’s description of RM systems as the ‘minimum form of life’ [25]. Furthermore, the hypothesis describes a ‘selfish RM system’, which contains at least two genes: coding for an MTase and REase. Here we show this ‘minimum form of life’ pushed to the extreme: just a single TspGWI N_473_A-coding gene replaces the ‘toxin-antitoxin’ system (cognate REase-MTase pair) [24]. In this work the DNA modifying site-specific ‘antitoxin’ (cognate MTase) is apparently functionally replaced by non-specific, cellular *E. coli* DNA damage repair systems. In accordance with the in vivo cultivation results, the TspGWI N_473_A expression experiments revealed low REase activity in vitro in crude recombinant *E. coli* extracts, despite the comparable amounts of both mutant and recombinant wt TspGWI proteins synthesized in vivo. Evaluating the phenomenon, we purified the TspGWI N_473_A [16] ( Fig. 2, see Methods and Table 1) and compared it with the recombinant wt TspGWI. Both recombinant enzymes were obtained essentially homogeneous, with minor contaminating bands visible only upon electrophoresis gel overloading. These preparations were further used for all analyses described in this paper (Fig. 2). While retaining the same general biochemical features, such as molecular size, isoelectric point, domain organization, pH, salt, magnesium ions requirements (not shown), owing to the single aa substitution, the REase activity of the TspGWI N_473_A variant (mutation within MTase domain) was barely detectable. Less than 0.8 % of the relative DNA cleavage activity was detected, in comparison to the recombinant wt TspGWI as assayed on the TspGWI-recognition site rich substrate—T7 DNA (Table 1; Fig. 3) or no activity was detected as assayed on the 390 bp PCR product containing two convergent TspGWI sites (Table 1; Fig. 4). The PCR fragment with two sites was selected to avoid a negative bias towards single site substrates, which we reported previously for TspGWI [11]. This result clearly shows the existence of intertwined communication and the mutual dependence of REase and MTase functions in the same polypeptide. In other words, REase and MTase functions can be uncoupled, but at the cost of greatly reduced REase activity. Equally surprising was the effect of the natural cofactor SAM and its analogue SIN on the REase activity of TspGWI N_473_A. SAM, a natural *Thermus* sp. enzymes family cofactor, has the least effect on wt TspGWI of all the members tested to date [16, 17]. In fact, a slight inhibitory effect was observed with both native (*Thermus* sp. GW-isolated) and recombinant wt TspGWI, reflected by minor differences in the stable partial DNA cleavage pattern [16] (Fig. 3). Such an inhibitory effect is even more evident in this work, where non-saturating enzyme concentrations are used together with the 390 bp PCR product, which contains only two TspGWI sites (Fig. 4).This subtle effect leads to an important conclusion that although the recombinant wt TspGWI protein apparently retained the ability to physically bind SAM, the interaction lost its functionality, so the REase activity could not be stimulated by the bound cofactor. Extending the reaction time does not cause SAM to have any further effect (stimulation) on wt TspGWI [16]. On the other hand, the MTase of the wt TspGWI is functional. This raises the question of whether the TspGWI protein has two separate SAM-binding pockets or whether binding SAM by the MTase moiety causes conformational changes, sending a signal along the polypeptide to the REase moiety. It is interesting that the very closely related TaqII REase from the same subfamily (but recognizing a different DNA sequence, thus not being a TspGWI isoschizomer) is very strongly (positively) affected by SAM [12]. The effect of SIN is similar in the case of the mutant TspGWI N_473_A REase. Besides reporting the ‘minimal’ case of the ‘minimum form of life’ phenomenon, the major novelty of this work lies in the description of the SIN effect on the TspGWI N_473_A enzyme variant—phenotypic mutation suppression resulting in the reactivation of the cleavage function. Wt TspGWI however responds to SIN with a DNA cleavage specificity change and minor stimulation. We reported previously that to enhance the effect of SIN-induced TspGWI specificity relaxation to a 3-bp REase, a molar excess of the enzyme/recognition site ratio and a prolonged incubation time had to be used [11]. Here, even when SIN is used under enzyme non-saturating (no enzyme excess) conditions (precisely like SAM: see Figs. 3, 4; Table 1), it has a striking effect on DNA cleavage restoration of TspGWI N_473_A as compared to the wt TspGWI protein, though depending on the DNA substrate to a different extent (Figs. 3, 4). Although SIN changes the specificity of wt TspGWI, it does not speed up the DNA digestion reaction (Figs. 3, 4) [11, 16] (this work). Surprisingly, however, it nearly completely suppresses the N_473_A mutation phenotype, resulting in an increase in TspGWI N_473_A activity to over 25–50 % of that of wt TspGWI REase, depending on the substrate used (Fig. 3, 4; Table 1). The overall stimulation of the TspGWI N_473_A mutant by cofactor analogue SIN is determined as a range of multiplicity rather than a precise number, owing to the limitations of the REase assay and the gel staining method used. Nevertheless, SIN-induced TspGWI N_473_A restriction activity restoration was clearly observed, with an app. 128-fold activity increase compared to the reaction without SIN supplementation (Fig. 3d–f), and with multiple T7 substrate DNA recognition sites. Under these conditions (the presence of SIN, T7 substrate DNA), moreover, TspGWI N_473_A REase exhibited app. 50 % of the activity of the wt TspGWI enzyme. In the case of the 390 bp DNA substrate the stimulation factor could not be calculated, as there was no DNA cleavage by TspGWI N_473_A in the absence of SIN (Fig. 4d). The addition of SIN resulted in the appearance of the expected DNA digestion products (Fig. 4f, lane 1), with an intensity corresponding to the partial cleavage pattern observed for wt TspGWI (Fig. 4a, lane 3).The relative specific activity of TspGWI N_473_A REase for the PCR fragment (two convergent recognition sequences) in the presence of SIN was therefore estimated at app. 25 % of wt TspGWI REase activity. The differences observed for T7 DNA and PCR product substrates could be attributed to the linear diffusion factor effect, as T7 DNA is app. 100 times longer than the PCR product and contains multiple TspGWI sites. To our knowledge, this is the first example of cofactor analogue-induced mutant REase activity restoration among REases and possibly among other SAM-utilizing enzymes. We hypothesize that the TspGWI N_473_A mutant has a more relaxed tertiary structure, with DNA cleavage catalytic aa residues shifted away from optimal positions. The SIN effect would act as a ‘molecular staple’, physically stabilizing aa residues within a SAM-binding pocket, thus restoring its competence to send conformational signals along the TspGWI N_473_A mutant polypeptide to the DNA scission catalytic centre, which has not been directly damaged by a distant mutation in the *tspGWIRM* gene section, coding for the NPPY methylation motif. The experiment shown in Fig. 5 indirectly confirms this conclusion. Both the wt TspGWI and TspGWI N_473_A mutant were used in digestions under titrated down concentrations of SIN. Substantial differences have been observed: wt TspGWI activity only slightly decreases over a very wide SIN concentration range (375 nM–100 μM), while TspGWI N_473_A restored activity drops dramatically much sooner (below 0.1 μM). Comparison of the digestion extent in Fig. 5a and Fig. 5b shows approximately the same partial digestion in lane 10 of Fig. 5b (TspGWI N_473_A) as in the lowest SIN concentration tested for wt TspGWI (Fig. 5a, lane 19). However, this comparison is affected by the wt TspGWI capability to digest DNA in the absence of SIN, with a 5-bp cognate recognition site specificity [15]. In the presence of SIN, wt TspGWI cuts DNA with a mixed specificity, e.g. 5-bp 5′-ACGGA-3′ [15] and 3-bp SIN-generated ‘affinity star’ specificity [11]. Thus, TspGWI N_473_A variant is a good model for ‘isolated’ analysis of the cofactor analogue effect on the REase, proving the crucial role of SIN binding in stabilizing cleavage-competent TspGWI N_473_A molecules. These experimental data may lead to a further conclusion that in ‘real life’ (no SIN preset) cellular environment, SAM stimulation of the undamaged *Thermus* sp. family wt enzymes is similar to that of TspGWI N_473_A by SIN. As this cofactor analogue is not a methyl group donor and it is anticipated that it has no affinity for the DNA cleavage motif PD-(D/E)XK, the interaction is allosteric, acting from a distant SAM(SIN) binding motif. The experiments shown in Figs. 3, 4, 5 shed some light on both activity modulation and specificity transition (wt TspGWI) and activity reactivation (TspGWI N_473_A) mechanisms. Because at the lowest activating concentrations of SIN, only a fraction of TspGWI N_473_A variant molecules (probably wt TspGWI as well, but the presence of the 5-bp cognate specificity complicates experimental interpretation) would form a complex with SIN (Fig. 5b, lanes 12–14), the possible activation scenario would need to include rapid diffusion of a relatively small SIN molecule away from the labile enzymes-SIN complexes in two putative modes: (i) from DNA bound TspGWI N_473_A(TspGWI)—SIN complexes, while remaining DNA-TspGWI N_473_A(TspGWI) complexes would retain a cleavage activation state or (ii) the transiently SIN-complexed TspGWI N_473_A(TspGWI) proteins would reach activation state while in a solution and remain activated after SIN dissociation, until DNA cleavage occurs. Both scenarios assume a multi turnover ‘catalytic’ action of SIN(SAM) on TspGWI enzymes, changing their polypeptide folding status. The discussion above did not account for a chemical binding constant value, as it has not been determined. Nevertheless, the binding constant rules that even at stoichiometric concentration of SIN and the TspGWI variants, not all of them will form complexes, further reinforcing the conclusions drawn above, concerning the need for SIN to transiently bind and activate the protein molecules. Alternatively, one can explain the sub-stoichiometric stimulation by the presence of mostly inactive protein molecules within the TspGWI variants preparations used. Nevertheless, the isolation protocols used gentle purification methods and the proteins are thermostable, thus we expect their high stability. During storage of the purified preparation for over a year at −20 °C, no activity losses were observed (not shown).

**Table 1:** Relative specific activities comparison of wt TspGWI and TspGWI N_473_A variant in the presence or absence of SAM or SIN

	Relative specific activity of DNA cleavage (%)					
	Bacteriophage T7 DNA (90 recognition sequences per DNA molecule) initial molar ratio of enzyme to recognition site 4.2:1			390 bp PCR fragment (2 convergent recognition sequences per DNA molecule) initial molar ratio of enzyme to recognition site 1.82:1		
REase	Without cofactor	SAM	SIN	Without cofactor	SAM	SIN
wt TspGWI (recombinant)	100 %	100 %	100 %	100 %	12.5–25 %	100 %
TspGWI APPY (N_473_A) mutant	Trace activity (less than 0.8 %)	Trace activity (less than 0.8 %)	50 %	No activity	No activity	25 %

The reactivation phenomenon also raises the question whether the restored DNA cleavage activity of the TspGWI N_473_A mutant remains the same as the wt TspGWI scission specificity. To address this problem we have generated two PCR substrates of 497 bp, based on an extension of the 390 bp substrate, evaluated in Fig. 4. The substrate extension has been done with longer primers for the purpose of precise determination of a digestion product’s length using polyacrylamide gel electrophoresis. Comparative digestion of a 497 bp substrate with two 5′-ACGGA-3′ cognate sites and a 497 bp substrate with no TspGWI recognition sites has shown that: (i) the cleavage pattern of wt TspGWI and TspGWI N_473_A is the same and (ii) the control substrate without 5′-ACGGA-3′ cognate sites is not digested by neither the wt TsoI nor TspGWI N_473_A mutant (Fig. 6). Taken together, these digestions show that reactivated TspGWI N_473_A mutant specificity remains the same as wt TspGWI.

In the experiments described in this publication, a partial DNA cleavage (a frequent feature of other subtype IIC/IIG enzymes) hindered the quantitative evaluation of the REase activity, in addition to the somewhat subjective nature of the REases activity assay. For comparing specific activities we decided to use relative values (obtained in a series of experiments, exemplified in Fig. 3a–f) rather than the classic specific activity units used for completely cleaving REases, owing to the partially subjective nature of REase unit estimation and the inability of *Thermus* sp. family enzymes to cleave substrate DNA completely. Series of photographs of gels segments as well as single DNA bands were scanned using UN-SCAN IT GEL software, taken with various exposure times and cross-calibrated baselines/intensities. The black arrows in Fig. 3 and Fig. 4 indicate the lanes of estimated identical or nearly identical extent of DNA digestion. The red arrows in Fig. 3 indicate the minimum amounts of the enzyme sufficient to obtain a stable partial digestion pattern. Despite a certain error and quantitative analysis limitations, Figs. 3, 4, 5 and 6 undoubtedly show the novel DNA cleavage activity reactivation phenomenon described. We believe that these findings, besides extending basic knowledge of REase-cofactor-DNA interaction, will also serve as a more general method for the alterations of specificities, activities and fidelities of IIS/IIC/IIG enzymes. Targeting a SAM-binding pocket or catalytic motifs of MTase with site-directed mutagenesis, followed by investigation of SAM and its analogues influence on the activity of the obtained enzyme variants, may bring novel, artificial REase prototype specificities not existing in nature or improve the cleavage characteristics of existing enzymes for DNA manipulation purposes. Moreover, the method could be used for the functional conversion of a substantial number of prototypes already found, which, however, are not used in gene cloning methodology because of their low Fidelity Index [12, 25] or partial cleavage feature.

## Conclusions

(i) The TspGWI N_473_A protein variant with altered methylation of the NPPY motif was expressed and isolated. Under standard reaction conditions it exhibits no MTase or trace REase activities (less than 0.8 % of DNA cleavage of wt TspGWI).
(ii) SIN—the SAM analogue with an inverted charge—stimulates impaired TspGWI N_473_A mutant and reactivates its cleavage function, at a level of 25–50 % activity of the wt protein.
(iii) The SIN-mediated phenotypic mutation suppression effect is the first described to date. It indicates that even though the REase and MTase functions reside in distant portions of the TspGWI polypeptide, intense interdomain communication is still taking place.
(iv) The TspGWI N_473_A mutant clone exercises a strong selection pressure on a recombinant *E. coli* host, causing long filaments formation and apparently following, further minimized, Kobayashi’s ‘minimum form of life’ hypothesis [23, 24].
(v) The method for changing the characteristics of IIS/IIC/IIG enzyme is more general and may serve as a tool for DNA recognition specificity and fidelity engineering.
